# Effectiveness of Heparin during Long-Term Tocolysis

**DOI:** 10.1155/2013/650532

**Published:** 2013-03-27

**Authors:** Tetsunori Inagaki, Shintaro Makino, Takashi Yorifuji, Motoi Sugimura, Satoru Takeda

**Affiliations:** Department of Obstetrics and Gynecology, Juntendo University School of Medicine, 2-1-1 Hongo, Bunkyo-ku, Tokyo 113-8421, Japan

## Abstract

*Objective*. Drip infusion during long-term tocolysis causes mechanical and infectious vasculitis and increases the frequency of peripheral venous catheter exchange (PVC), thereby placing a burden on patients. Our study aim is to confirm whether heparin ameliorates pain due to vasculitis during long-term tocolysis and reduces the frequency of peripheral venous catheter exchange. *Design*. Prospective study. *Setting and Sample*. All the patients requiring admission because of the presence of uterine contraction or progressive cervical dilatation from August 2009 to June 2011 at Juntendo University in Japan. *Methods*. Heparin was used for patients at the time the total number of peripheral venous catheter exchanges exceeded 5 in two weeks, and we evaluated whether heparin reduced the frequency of peripheral venous catheter exchange and improved the visual analog scale (VAS) for patients. The main outcome measures frequency of PVC exchange and VAS. *Results*. This study demonstrated that heparin reduced the frequency of peripheral venous catheter exchange (*P* = 0.0069) and VAS (*P* = 0.042). No side effects were noted. *Conclusion*. Heparin could satisfy patients during long-term tocolysis in terms of ameliorating pain due to vasculitis and reducing the PVC exchange frequency.

## 1. Introduction

Preterm labor is considered differently in Japan and USA. Continuous tocolysis for more than 48 hours is not recommended in the USA. But in Japan Ritodrine hydrochloride is usually used for long-term tocolysis under the presence of uterine contraction or progressive cervical dilatation. This therapy leads to the lower premature delivery rate in Japan (actually 5.7% in 2007) than the other country (10.6% in north America). But drip infusion therapy during long term tocolysis causes pain due to vasculitis, and the PVC must be exchanged often.

Three kinds of vasculitis mechanism have recently been proposed [1]. One is chemical, another is mechanical, and the third is bacterial vasculitis. Chemical vasculitis is caused by hypertonic infusion. The pH discrepancy between the infusion and inside blood vessels is also a cause of vasculitis. The peripheral venous catheter itself also causes vasculitis by injuring endothelial cells, a form of mechanical vasculitis. Infection with bacteria also causes vasculitis. These three elements can combine to exacerbate vasculitis during long term tocolysis. These elements are thought to be prevented by heparin's anti-inflammatory [2], anticoagulant [3, 4], and cell migration effects. Heparin's anti-coagulant effect can improve blood flow. Furthermore, cell migration is induced to repair endothelial cells. Heparin also prevents both vessel fibrosis and neutrophil gathering at inflammatory sites [5]. Many retrospective and prospective studies [6–10] confirmed the effect of heparin against vasculitis but few research is done against pregnant women. As we paid attention to these effects, heparin was injected with Ritodrine into the 5% glucose bottle during drip infusion. By reducing pain and the frequency of peripheral venous catheter exchanges, patients' satisfaction during hospitalization can be improved. Heparin's effect during long term tocolysis was assessed prospectively in this study.

## 2. Methods

The observation period was from August 2009 to June 2011. We obtained a written informed consent from patients. All patients required admission for long term tocolysis were eligible for enrollment in this prospective study, if consent was obtained.

Vasculitis increases the pain, where peripheral venous catheter (PVC) placed, and the frequency of PVC exchange. So we used PVC exchange frequency and VAS of the patients to assess the objective scale in this study.

**Figure 1:**
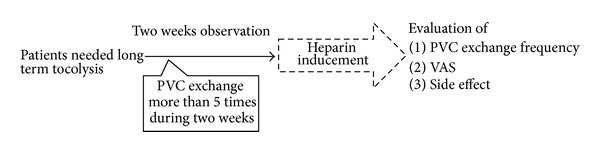
Dose of heparin needed for treatment. The figure shows the procedure of this study. All the patients needed tocolysis can be entry for this study. During two-week observation period, if PVC exchange frequency reaches over 5 times, heparin is induced to the patients. Study parameters are peripheral venous Catheter exchange frequency, Visual analogue scale and side effect of heparin.

 The frequency of PVC exchange was counted for two weeks after drip infusion tocolysis being started in admission. When PVC exchange exceeded 5 times in two weeks, heparin was administered with patient consent. We used the PVC, (the Sure Seald SHURflo II, made by Terumo Corporation, Tokyo, Japan). The amount of heparin in the bottle was set at almost 6000 IU per day. The precise amount regulated by the velocity of drip infusion is described in Table 1. After induction, we evaluated the PVC exchange frequency, VAS, and blood examination results (platelets, liver enzymes, and coagulation system). The precise protocol of this study is described in Figure 1. For the statistical analysis, Welch's *t*-test was selected and a predictive value <0.05 was regarded as indicating a statistically significant difference. 

## 3. Results

**Figure 2:**
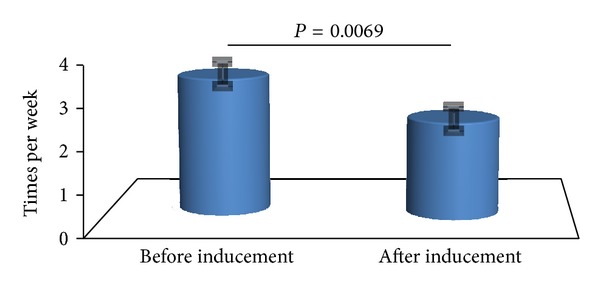
Before inducement versus after inducement of heparin PVC exchange frequency. The frequency of PVC exchange reached to 3.32 ± 0.28 per week before heparin inducement. After inducement, the number of PVC exchange reduced to 2.34 ± 0.23 per week (*P* = 0.0069).

The total number of patients enrolled in this study was 45. Adaptations for tocolysis are described in Table 2. For the first 2 weeks, the frequency of PVC exchange exceeded 5 times in 30 patients, and the rest 15 patients also needed the heparin rescue in more two weeks. Patient backgrounds are presented in Table 3. 27 patients were primipara and 18 were multipara. Three twin pregnancies were included. The average gestation when drip infusion started was about 26.0 ± 1.4 weeks and heparin was induced at 29.2 ± 1.2 weeks. For the first two weeks, the frequency of peripheral venous catheter exchange was 3.32 ± 0.28 times per week. After heparin inducement, the number of peripheral venous catheter exchanges was reduced to 2.34 ± 0.23 (*P* = 0.0069).There was a significant difference in the frequency of PVC exchange between before and after inducement of heparin (Figure 2). 

**Figure 3:**
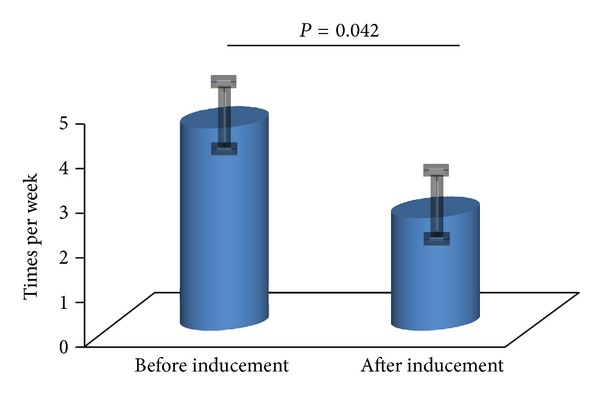
Before inducement versus after inducement of heparin VAS evaluation. The VAS before heparin inducement was 4.5 ± 0.5 and improved to be 2.5 ± 0.5 after inducement. This VAS improvement shows that patients obviously felt better comfortable as to the drip infusion therapy after heparin inducement.

The VAS before induction was 4.5 ± 0.5 and improved to 2.5 ± 0.5 after induction (*P* = 0.042). There was a significant difference before versus after heparin rescue (Figure 3).

No drug-induced liver enzyme elevation or anti-coagulant abnormalities occurred (Table 4). The total average blood loss at delivery was 531 ± 297 mL, indicating no adverse effect of heparin usage. The birth outcomes are shown in Table 5. 

## 4. Discussion 

Our study confirmed heparin's beneficial effect on vasculitis. Patients during long term tocolysis are highly stressed due to the pain caused by the vasculitis. If heparin has an anti-inflammatory effect and ameliorates vasculitis, the patient's stress during admission can be improved.

Civelek et al. reported that low-molecular-weight heparin may adversely affect incisional wound healing by suppressing the early inflammatory process [11]. Lever et al. used size-fractioned heparin experimentally [5]. They assessed the effect on elastase release induced of formyl Met Leu Phe (fMLP) from neutrophils. Elastase release was inhibited by the very-low-molecular-weight fraction of heparin, while neutrophil endothelial adhesion was unaffected. Heparin's anti-inflammatory effect was examined in these reports. 

In the surgical field, peripheral parenteral nutrition (PPN) is used instead of total parenteral nutrition. With PPN, however, peripheral vein thrombophlebitis and vascular pain are problems. Inoue et al. reported that a 1 IU/mL heparin injection into the bottle reduced both vascular pain and the rate of peripheral venous catheter exchange [12]. 

A prospective study on prolonging the usability of peripherally placed percutaneous central venous catheters in neonates demonstrated the beneficial effects of heparin on these catheters [13–16].

Our study confirmed heparin's beneficial effect on the pain during long term tocolysis. By ameliorating inflammation and coagulation, patients can maintain the peripheral venous catheter less painful. During this study, most of our patients felt less painful after heparin induction. Heparin inducement can mitigate pain, and thereby improve VAS. 

In this study, neither drug induced hepatitis nor anti-coagulant abnormalities occurred. However, heparin induced thrombocytopenia (HIT) develops in approximately 0.5% of patients during heparin-induction therapy [17, 18]. In some cases of pregnancy, HIT were induced by heparinization [19–21]. Thus, we must conduct precise blood examinations during heparinization. 

The risk of heparin's side effects is unacceptable. However, the benefits of heparin should also be considered. The stress on patients during therapy is considerable. Even if complications such as HIT occur, simply discontinuing heparin is enough to achieve recovery.

Some groups have suggested that a lower dose of heparin than that of our study would be effective. Our study is the effective dose for curing coagulant abnormalities. Thus, a lower dose of heparin might be enough to improve the pain. The next survey must consider the lower effective dose of heparin. Furthermore, a larger subject number and in vitro experiments would be needed for the next survey to prove that heparin ameliorates vasculitis itself in patients.

*Key Message.* Heparin can ameliorate the pain due to vasculitis during long term tocolysis and reduce the frequency of peripheral venous catheter exchange. This method lifts the burden from patients during admission.

## Figures and Tables

**Table 1:** Dose of heparin needed for treatment. The dose of heparin needed per bottle regulated by the velocity of drip infusion is described. Total dose of heparin per day is regulated to be about 6000 IU.

Velocity	Heparin per bottle	Total dose per day
20 mL/h	6000 IU	5760 IU/day
25 mL/h	5000 IU	6000 IU/day
30 mL/h	4000 IU	5760 IU/day
35 mL/h	3500 IU	5880 IU/day
40 mL/h	3000 IU	5760 IU/day

**Table 2:** Reasons for tocolysis. The reason for Ritodrine tocolysis is mostly because of the prevention of premature delivery. One case is used for the control of fetus heat rate because of fetal AV block.

Adaptation for tocolysis	
Progressive cervical dilatation	39
Previa	1
Polyamnios	4
Fetal AV block	1

**Table 3:** background of the patients. The patients' background is described. Most of the patients needed the Ritodrine tocolysis until around 36 weeks of gestation because of the preterm uterine contraction or the progressive cervical dilatation.

Background	
Age	38 ± 2.2
Pri/para	27/18
Single/twin	42/3
Gestation of Ritodrine inducement	26.4 ± 1.4
Gestation of heparin inducement	29.2 ± 1.2
Gestation tocolysis finished	34 ± 1.8

**Table 4:** Side effect of heparin. For the evaluation of side effect of heparin, blood examination was performed. Most of the patients showed no obvious elevation of liver enzyme, no thrombocytopenia, and no prolongation of APTT.

	Before heparin inducement	After heparin inducement	*P* value
Plate (×10^3^)	25.1 ± 5.9	24.7 ± 6.5	0.787
AST (IU)	17.3 ± 2.0	18.1 ± 5.0	0.706
ALT (IU)	12.3 ± 4.9	14.2 ± 1.3	0.787
APTT %	33.3 ± 2.4	33.9 ± 2.4	0.273

**Table 5:** Birth outcome. Average gestation at birth is earlier because of the background of premature delivery. But nothing was influenced by the inducement of heparin.

Outcome	
Gestation at birth	36 ± 2.8 weeks
Birth weight	2468 ± 665 g
pH of umbilical artery	7.33 ± 0.08
Apgar score (1/5)	8.1 ± 0.7/9.1 ± 0.9

## Abbreviation

VAS: visual analog scale
PVC: peripheral venous catheter.
